# Enema Rashes

**Published:** 1902-01-18

**Authors:** 


### ENEMA RASHES.

As is well known the administration of enemata is apt in certain cases to be followed by irritable rashes of the nature of erythema or urticaria. These have long been regarded as of toxic origin, it being imagined that where fæcal masses have been allowed to accumulate their decomposition has led to the production of toxic matters which on being dissolved are absorbed into the circulation and set up various disturbances. When these rashes are met with in large institutions, such as general or children's hospitals, they are liable to cause considerable anxiety, since they may readily be confounded with certain other eruptions, such as those of scarlet fever or German measles, drug rashes, septic rashes, and those arising in the course of serum treatment. Dr. Charles Bolton,[1] resident medical officer, University College Hospital, points out that the occurrence of these rashes seems to have some relation to the kind of soap which is employed in forming the enema. He says that having been informed by a nurse that in the hospital where she was trained soft soap was used, and that enema rashes were never seen, he determined to put the matter to the test. With this object in January, 1901, he ordered soft soap to be used for the enemas in one-half of the medical and surgical wards of the hospital and hard yellow soap in the remaining half of the wards. This order continued in force for six months, during which time a total of 903 enemas were given to 500 patients. Of these, 407 soft soap enemas were given to 234 patients, and 496 hard soap enemas to 266 patients. In the latter series 17 rashes resulted. but in the former he did not find a single one. Of the 17 rashes never more than one occurred in the same person, and when more than one enema was given the rash followed the first, second, third, or fourth, but not the later ones. In one case, after a patient had received several soft-soap enemas, the mistake was made of giving him one compounded with hard soap, when a rash resulted. All the rashes appeared on the day following the injection, and their duration was from one to three days. It is a very interesting observation. Our readers will of course remember that hard soap is made with soda, and soft soap with potash, and one may be inclined to jump to the conclusion that the different effects noted may have had to do with the different forms of alkali contained in the two soaps, but it is possible that differences in the other ingredients may have had something to do with the matter. Yellow soap is made, we believe, from tallow or palm-oil, it contains rosin, and most of the glycerine is removed from it in the course of manufacture. Soft soap, on the contrary, is said to be made from linseed or other oil, and contains all the glycerine which is set free in its production; which may perchance have some bearing on the problem.

[1] Clinical Jour., Jan. 1.

